# Spatiotemporal resolution in high-speed atomic force microscopy for studying biological macromolecules in action

**DOI:** 10.1093/jmicro/dfad011

**Published:** 2023-02-06

**Authors:** Kenichi Umeda, Steven J McArthur, Noriyuki Kodera

**Affiliations:** Nano Life Science Institute (WPI-NanoLSI), Kanazawa University, Kakuma-machi, Kanazawa 920-1192, Japan; Nano Life Science Institute (WPI-NanoLSI), Kanazawa University, Kakuma-machi, Kanazawa 920-1192, Japan; Nano Life Science Institute (WPI-NanoLSI), Kanazawa University, Kakuma-machi, Kanazawa 920-1192, Japan

**Keywords:** atomic force microscopy, single-molecule imaging, biophysics, proteins, nucleic acids, biomolecules

## Abstract

High-speed atomic force microscopy (HS-AFM) is a unique approach that allows direct real-time visualization of biological macromolecules in action under near-physiological conditions, without any chemical labeling. Typically, the temporal resolution is sub-100 ms, and the spatial resolution is 2–3 nm in the lateral direction and ∼0.1 nm in the vertical direction. A wide range of biomolecular systems and their dynamic processes have been studied by HS-AFM, providing deep mechanistic insights into how biomolecules function. However, the level of mechanistic detail gleaned from an HS-AFM experiment critically depends on the spatiotemporal resolution of the system. In this review article, we explain the principle of HS-AFM and describe how the resolution is determined. We also discuss recent attempts to improve the resolution of HS-AFM to further extend the observable range of biological phenomena.

## Introduction

Atomic force microscopy (AFM) is a palpation-type microscopy that creates three-dimensional maps of sample surfaces at a high spatial resolution by scanning a tiny probe tip attached to the end of a cantilever over a surface [1]. Since AFM measures the forces acting between the probe tip and the sample surface, AFM can observe a wide range of samples in various environments. Thus, AFM is now routinely used to characterize the topographies and mechanical properties of samples and continually pushes the frontiers of nanoscience and nanotechnology in physics, chemistry and biology [2–4].

AFM holds a unique significance in biological sciences because it is the only microscopy that can directly capture submolecular-resolution images of biological samples under near-physiological conditions without sample staining or chemical labeling [5–7]. In addition, AFM has been used for force measurements to estimate the strength of intra- and inter-molecular bonds at the single-molecule level [8–10] and the mechanical properties of living cells [11,12]. These applications have led AFM to become recognized as a multifunctional toolbox for biology [2]. However, the effective temporal resolution of conventional AFM is insufficient to capture dynamic behaviors of biological samples, requiring at least 30 s to obtain a single image. As a result, molecules moving on the substrate surface appear to be blurred or are not visible at all. This drawback had previously limited the application of AFM in functional studies of biological molecules.

To overcome this limitation, high-speed AFM (HS-AFM) for biological applications has been developed. After initial prototyping [13,14] and extensive improvements [15–19], HS-AFM was first put into practical use around 2008 [20]. While the temporal resolution depends on the sample to be imaged and the imaging conditions, current HS-AFM systems can take successive images of biological samples ≤100 ms, allowing clear imaging of sub-100 ms molecular movements and dynamic processes. Importantly, despite the rapid pace of interactions between the probe tip and the sample, invasiveness is sufficiently low that the sample remains structurally and dynamically intact even after prolonged imaging.

Owing to the high speed and low invasiveness of HS-AFM, dynamic structural changes and functional processes in various biomolecular systems have been successfully imaged using HS-AFM, as reviewed previously [21–24]. In the past 3 years alone, numerous representative results have been reported on the following biomolecular systems: transmembrane proteins [25–30], peripheral membrane proteins [31–33], cytoskeletons and their binding proteins [34–38], nucleic acids and their binding proteins [39–52], intrinsically disordered proteins [53–57], molecular motors [58–61], amyloidogenic proteins [55,62–64], protein assemblies [65–68], membrane vesicles [69–71], disruption of cellular membrane [72–75], membraneless organelles [76,77] and artificial molecules [78–82]. The remarkable breadth of these achievements showcases the power of HS-AFM to open new avenues of investigation into diverse biomolecular systems and to obtain unique dynamic and mechanistic insights about these systems.

Although beyond the scope of this review, AFM operated in frequency modulation mode (FM-AFM) [83] has achieved atomic-scale imaging [84,85], atom manipulation [86,87] and chemical identification [88] for solid samples in ultra-high vacuum. Remarkably, true atomic-resolution imaging was demonstrated in liquid by FM-AFM [89,90], although applications toward biological molecules are still limited and the temporal resolution is not as high as that of HS-AFM [91–93]. The spatiotemporal resolution of FM-AFM is discussed in detail in previous studies [94–96].

In this review, we focus on HS-AFM for imaging biological macromolecules in action. First, we describe the principles underlying HS-AFM measurement and performance. Then, we explain the concept of the spatiotemporal resolution of HS-AFM. Finally, we discuss the recent attempts to improve the resolution of HS-AFM to extend its application range in the biological sciences.

### Principle of HS-AFM

In brief, all AFM systems capture the topographies and physico-chemical properties of sample surfaces by scanning a probe tip attached to the free end of a microcantilever over a sample. While AFM can be performed in a number of operational modes [2], HS-AFM specifically employs the tapping mode, also called amplitude modulation mode or intermittent contact mode [97], which is usually used to observe fragile biological samples.

**Fig. 1.**
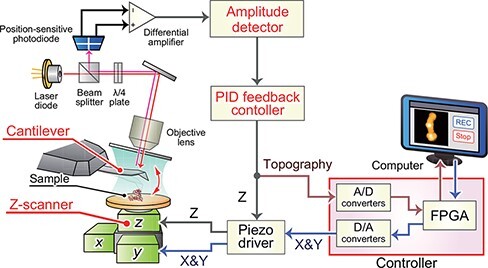
Diagram of HS-AFM system.

The system configuration of HS-AFM is basically the same as that of conventional tapping-mode AFM, but all components in the system are optimized for high-speed imaging (Fig. 1). In the tapping mode, the cantilever oscillates in the Z direction at around its first resonance frequency. When the cantilever approaches a sample surface, the oscillating probe tip intermittently taps the sample surface at the bottom of its swing. Hence, the oscillation amplitude of the cantilever varies with the degree of contact. Using an optical beam deflection (OBD) system [98,99], the cantilever deflection is monitored by a laser beam reflected from the cantilever and guided to a position-sensitive photodiode. The output signals from the photodiode are conditioned to provide a differential signal corresponding to the cantilever deflection. The cantilever deflection signal is then converted to an amplitude signal by the amplitude detector. After that, the measured amplitude signal is compared with the preset amplitude values (i.e. the feedback set point amplitude), and their difference signal (i.e. the error signal) is fed into a proportional–integral–derivative (PID) feedback controller. The output signal from the feedback controller is fed into a Z piezo driver which displaces the Z-scanner, onto which the sample stage is affixed, in the Z direction so that the error signal approaches zero.

HS-AFM, high-speed atomic force microscopy.

This series of operations is continuously repeated at different points on the sample surface during lateral scanning of the sample stage in the XY directions. The force acting between the probe tip and the sample surface is kept constant, minimizing the disruptive effect of the probe on the sample. The XY scanning signals are generated from a digital-to-analog (D/A) converter, and the output signal from the feedback controller at each XY position is recorded by an analog-to-digital (A/D) converter. This allows reconstruction of the topographic image of the sample surface. The temporal resolution of AFM is improved by decreasing the time required for each loop.

### Feedback performance of HS-AFM

As mentioned earlier, two requirements must be met simultaneously to successfully apply HS-AFM to biological samples. One is temporal resolution high enough to capture the dynamic behavior of the sample, and the other is to minimize invasiveness in order to avoid disruption and deterioration of the sample. To this end, the following mechanical and electronic devices have been developed: small cantilevers with a small spring constant *k*_c_ and a high resonance frequency *f*_c_ [14,15], an OBD detection system using an objective lens specialized for small cantilevers [14], a high-speed amplitude detector [14], high-speed scanners [14,16,100], an active damper for the scanners [18], a dynamic PID feedback controller that outputs a large signal only when the probe tip is detached from the sample surface [19] and a fast data acquisition system [17]. Details of these technical developments are described in previous studies [20,101].

The feedback system includes the cantilever, amplitude detector, PID feedback controller and Z-scanner (Fig. 1). The speed of the feedback control can be quantified by measuring the feedback bandwidth (*f*_B_) in the closed loop system. Usually, *f*_B_ is defined by the feedback frequency at which a phase delay of *π*/4 occurs between the sample surface features to be observed (i.e. disturbance signal) and the Z-scanner displacement (i.e. feedback signal).

**Fig. 2.**
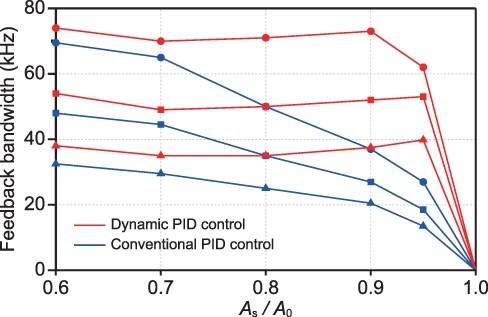
Feedback bandwidth *f*_B_ is affected by various factors. *f*_B_ was measured by changing *A*_s_/*A*_0_ and *A*_0_/*H*_s_ ratios using a mock AFM system [19]. Blue and red plots were obtained by conventional and dynamic PID controllers, respectively. Circle, square and triangle marks correspond to the *A*_0_/*H*_s_ ratios of 5, 2 and 1, respectively [19].

AFM, atomic force microscopy; PID, proportional–integral–derivative.

In reality, *f*_B_ is affected by various factors, including the ratio of the free oscillation amplitude of the cantilever *A*_0_ and the sample height *H*_s_, the ratio of *A*_0_ and the feedback set point amplitude *A*_s_ and the physico-chemical properties of the sample and the tip–sample convolution [19,102,103] (Fig. 2). However, when *H*_s_/*A*_0_ and *A*_s_/*A*_0_ are small enough, *f*_B_ is well approximated by an open loop system using the sum of the time delays of the above devices as


(1) $${f_{\rm{B}}} = \frac{1}{8}/\left( {{\tau _{\rm{c}}} + {\tau _{{\rm{amp}}}} + {\tau _{\rm{z}}} + {\tau _{\rm{d}}}} \right),$$

where *τ*_c_, *τ*_amp_ and *τ*_z_ are the response time for the cantilever, amplitude detector and Z-scanner, respectively, and *τ*_d_ is the sum of the other small time delays in the feedback system. *τ*_c_, *τ*_amp,_ and *τ*_z_ are further expressed by


(2) $${\tau _{\rm{c}}} = \frac{{{Q_{\rm{c}}}}}{{\pi {f_{\rm{c}}}}},$$

(3) $${\tau _{{\rm{amp}}}} = \frac{{\Delta \varphi }}{{2\pi {f_{\rm{c}}}}}$$

and


(4) $${\tau _{\rm{z}}} = \frac{{{Q_{\rm{z}}}}}{{\pi {f_{\rm{z}}}}},$$

where *f*_c_ (*f*_z_) and *Q*_c_ (*Q*_z_) are the resonance frequency and the quality factor of the cantilever (Z-scanner), respectively, and Δ*φ* is the phase delay in the amplitude detection with respect to the cantilever’s oscillation cycle.

The current HS-AFM system achieves *f*_B_ ∼ 70 kHz for low height samples [19], where *τ*_c_ is ∼0.4 µs using a cantilever with *f*_c_ ∼ 1.2 MHz and *Q*_c_ ∼ 1.5 in liquid, *τ*_amp_ is ∼0.4 µs using an amplitude detector with Δ*φ* ∼ *π* [14] and a cantilever with *f*_c_ ∼ 1.2 MHz, *τ*_z_ is ∼0.8 µs using a Z-scanner with *f*_z_ ∼ 200 kHz and *Q*_z_ ∼ 0.5, and *τ*_d_ is ∼0.1 µs. The scanning area of HS-AFM for the observation of biological molecules in the XY and Z directions is ∼2 × 2 µm and ∼0.5 µm, respectively. In contrast, although the scanning area of conventional AFMs is much larger than HS-AFM, they typically have *f*_B_ < 1 kHz.

### Temporal resolution of HS-AFM imaging

**Fig. 3.**
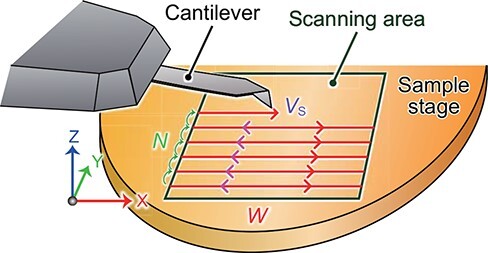
Imaging parameters that determine the temporal resolution of AFM.

To consider the temporal resolution of HS-AFM, which we define as the minimum time to obtain an image *T*_min_, we assume the XY-raster scanning method commonly used in biological applications (Fig. 3), notwithstanding non-raster scanning methods that have also been proposed [104,105]. When we suppose that an image is to be taken, given the smallest apparent width of features on the sample surface (*σ*), the scan range in the X direction (*W*) and the number of scanning lines in the Y direction (*N*), *T*_min_ is expressed by


(5) $${T_{{\rm{min}}}} = \frac{{\pi NW}}{{2\sigma {\theta _{{\rm{max}}}}{f_{\rm{B}}}}},$$

AFM, atomic force microscopy.

where *θ*_max_ (in radians) is the maximum possible phase delay in scanning the sample surface at which the resulting excess force exerted by the probe tip does not disturb the biological structure or function of the sample [106]. *σ* is approximated by the convolution of the probe tip radius *R*_t_ and the smallest sample feature radius to be observed *R*_s_ (i.e. $\sigma = \sqrt {{R_{\rm{t}}}{R_{\rm{s}}}} $).

In practice, the maximum scanning velocity of the probe tip *V*_max_ derived from *T*_min_ is a useful parameter for performing imaging experiments with optimized instrument performance. *V*_max_ is given by


(6) $${V_{{\rm{max}}}} = \frac{{2NW}}{{{T_{{\rm{min}}}}}}.$$

The line rate, which represents the X-scanning frequency, is another parameter that is often used to describe the imaging conditions of experiments. However, since the length of each X scan line varies depending on the size of the scan area, it would be more useful to report the scanning velocity of the probe tip *V*_s_, where *V*_s_ should be smaller than *V*_max_. Since the efficiency of the feedback response is directly related to *V*_s_, this allows the feedback response to be directly compared between studies regardless of the size of the scan area.

Although *θ*_max_ depends on the fragility of each target molecule, *θ*_max_ is estimated to be ∼*π*/9 (i.e. ∼20°) for proteins, according to previous imaging studies [107]. Under a typical condition for imaging structural proteins (e.g. *W* = 80 nm, *N* = 80, *σ* = 5 nm, *f*_B_ = 70 kHz and *θ*_max_ = *π*/9), *V*_max_ and *T*_min_ are estimated to be ∼150 μm/s and ∼82 ms which corresponds to an imaging rate of ∼12.2 frames per second (fps), respectively. Notably, some intrinsically disordered proteins with *σ* ∼ 2 nm can be imaged without deterioration of their morphological and dynamic features for >2000 frames, even with an image acquisition time *T* of 20 ms (50 fps), with *W* = 60 nm, *N* = 36 and *f*_B_ = 70 kHz. This suggests that HS-AFM imaging can be performed even under conditions where *θ*_max_ probably exceeds *π*/9. This may be due to the substantially low height of intrinsically disordered regions, equivalent to the diameter of a single polypeptide chain (i.e. ∼0.5 nm). Thus, higher temporal resolution imaging would be applicable to biological molecules with low height [108,109].

Importantly, Eq. (5) shows that the number of pixels in the X direction does not affect *T*_min_: that is, putting a large number of pixels in the X direction does not result in a decrease in temporal resolution. Indeed, some imaging studies skillfully used this idea to collect more information from the sample surface by using a rectangular pixel, with a smaller pixel size and a higher pixel count in the X direction [72,110,111]. Furthermore, Eq. (5) implies that the temporal resolution of AFM can be increased by decreasing *N*, as demonstrated previously [35,112,113]. More specifically, line scanning mode (*N* = 1) provides a temporal resolution as short as a few tenths of the time needed to capture a full image [13,114]. This approach has been demonstrated in the successful quantification of the fast kinetics of membrane proteins [115–117]. At the extreme, it is also possible to follow the height change at a certain fixed position on a sample [118,119]. Indeed, oligomeric states and concentrations of a membrane binding molecule on a biological model membrane have been studied by HS-AFM in this way at a temporal resolution of ∼10 μs [115].

### Spatial resolution of HS-AFM imaging

Since AFM topographic images depict the height variation in the Z direction as a function of the spatial coordinates in the XY plane, the spatial resolution must be separated into vertical (Z) and lateral (XY) resolution. These are affected by various factors: mechanical and electrical noise from the instrument, the size and shape of the probe tip, the forces acting between the probe tip and sample, the physico-chemical properties of the sample and so on [120]. Biological samples in particular need additional considerations because they have viscoelastic properties and are highly dynamic in nature, exhibiting thermal fluctuation and functional movements; these will be discussed later. However, for simplicity, we will first consider the case where the deformations and movements of the sample can be ignored.

The vertical resolution *δZ* is determined by the thermal noise of the cantilever *δZ*_th_, the noise of the OBD sensor *δZ*_det_ and the noise of the Z-scanner *δZ*_s_, when the cantilever is oscillated at or near its first resonance frequency. As these noise sources are independent, *δZ* is given as


(7) $$\delta Z = \sqrt {\delta {Z_{{\rm{th}}}}^2 + \delta {Z_{{\rm{det}}}}^2 + \delta {Z_{\rm{s}}}^2} .$$

Since *δZ*_det_ and *δZ*_s_ are negligible compared to *δZ*_th_ in the HS-AFM system, Eq. (7) can be approximated as


(8) $$\delta Z \cong \sqrt {\frac{{4{k_{\rm{B}}}{T_{{\rm{exp}}}}{Q_{\rm{c}}}{f_{\rm{B}}}}}{{\pi {f_{\rm{c}}}{k_{\rm{c}}}}}} $$

where *k*_B_ and *T*_exp_ are the Boltzmann constant and the temperature in Kelvin during the experiment, respectively [121]. *δZ*_th_ is estimated to be ∼0.05 nm in our HS-AFM system under typical parameters (i.e. *f*_c_ = 1.2 MHz, *k*_c_ = 0.2 N/m, *Q*_c_ = 1.5, *f*_B_ = 70 kHz and *T*_exp_ = 298 K). This is a sufficient vertical resolution to observe the 0.34-nm layer-to-layer step height of highly oriented pyrolytic graphite, corresponding to the average size of amino acids [54].

**Fig. 4.**
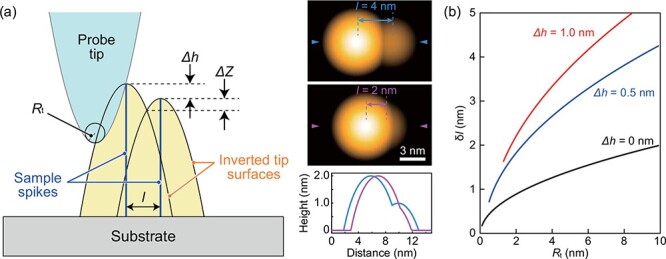
Estimation of lateral spatial resolution of AFM based on a simple geometric configuration. (a) Left, AFM image reconstruction based on the Rayleigh criterion. Two sample spikes are scanned by a parabolic probe tip with radius *R*_t_. Modified based on ref [122]. Right, pseudo AFM images for the two spikes (top and center) and their cross-sections (bottom). The two spikes, separated by 4 nm (top) and 2 nm (center), are scanned by a probe tip with *R*_t_ of 5 nm. The height difference Δ*h* of the spikes is 1 nm. Cross-sections are along the axis denoted by the arrowheads, which passes through the peaks. (b) Lateral resolution of HS-AFM *δl* as a function of *R*_t_ for two adjacent features, assuming *δZ* of 0.05 nm. Modified based on ref [120].

AFM, atomic force microscopy; HS-AFM, high-speed atomic force microscopy.


The lateral resolution is considered using the Rayleigh criterion and is predominantly determined by the shape of the probe tip [122]. As shown in Fig. 4a, a pair of sharp spikes, with a height difference Δ*h*, separated by distance *l* is scanned by a probe with a tip radius *R*_t_. As the samples are considerably sharper than the tip, the resulting topographic image shows a pair of inverted probe tips intersecting the top of the spikes. The intersection of the shapes of the inverted probe tips forms a small dimple with depth Δ*Z*. The lateral resolution *δl* can be determined by the minimum lateral separation *l* for which Δ*Z* is larger than the vertical resolution of the system, *δZ*. Therefore, *δl* can be approximated as

(11) $$\delta l \cong \sqrt {2{R_{\rm{t}}}} \left( {\sqrt {\delta Z} + \sqrt {\delta Z + \Delta h} } \right),$$

for $\delta l \gt \sqrt {2{R_{\rm{t}}}\Delta h} $, where Δ*Z* and Δ*h* are finite quantities, and thus their multiplications can be ignored. Figure 4b shows the lateral resolution as a function of *R*_t_ using *δZ* of 0.05 nm as estimated earlier. When samples have no height difference, *δl* of ∼1.5 nm can be achieved with a probe of *R*_t_ ∼ 5 nm. By contrast, in a realistically observable case where samples have a height difference of only ∼1 nm, *δl* increases to 3–4 nm using the same probe tip of *R*_t_ ∼ 5 nm. These probe tips can be fabricated at the end of a cantilever using electron beam deposition followed by argon plasma etching [22]. In good cases, *δl* as low as ∼1 nm can be achieved for samples with low height differences [29,109], implying that probe tips can be fabricated using this procedure with *R*_t_ as low as 1–2 nm.

Since AFM images are digital data, the spatial resolution is also affected by the data acquisition process. According to the sampling theorem, the size of a single image pixel should be less than half of the desired resolution. The A/D and D/A converters used in our current HS-AFM system are 12 bits for ±1 V (PCI-3525 and PCI-3305, Interface, Japan) corresponding to minimum digital resolutions of ∼0.10 nm and ∼0.03 nm in the XY and Z directions, respectively. While the minimum digital resolutions depend on the maximum displacements of the scanner, they are smaller than the lateral and vertical resolutions of the instrument.

In principle, AFM generates accurate and reliable height information relative to a reference plane. However, as mentioned earlier, the width and volume of an imaged object should be carefully quantified because AFM images are acquired with a convolution effect caused by the tip scanning [123]; this effect is especially pronounced for biological molecules whose size is comparable to *R*_t_. In addition, when observing objects with sizes similar to *R*_t_, a multi-tip effect often appears in images [124]. Both effects must be taken into account when analyzing and interpreting width and volume data obtained by AFM.

Remarkably, to overcome the limit on AFM spatial resolution imposed by the tip convolution effect, a method called the localization image reconstruction algorithm [125] was developed based on the localization microscopy technique that has revolutionized the spatial resolution of fluorescence microscopy [126,127]. This method has since been applied to demonstrate that single amino acid residues on membrane proteins in native and dynamic conditions can be resolved by AFM [29,117,125].

### Apparent spatial resolution and data asynchronicity

**Fig. 5.**
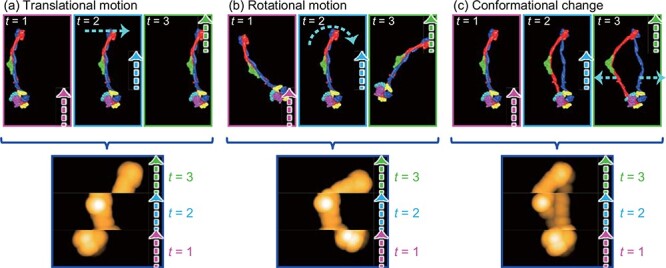
Effect of data asynchronicity on AFM imaging for a mobile sample. (a) Translational motion, (b) rotational motion and (c) conformational change of a molecule of SMC5/6 complex over time (*t* = 1, 2, 3). The thick dashed lines represent the progress of the scan whereas the thin dashed lines represent the protein movements. The upper and lower panels show the shape of the molecule at each time and the single AFM images taken over *t* from 0 to 3, respectively. The AFM images are pseudo AFM images based on the Protein Data Bank structure (PDB 7QCD), generated using an AFM simulator.

Next, we consider the case where the sample deforms and moves during AFM imaging. It is important to consider that the data for all pixels in an image are not acquired at the same time, but at different times during the lateral scanning of the sample stage; this is called data asynchronicity. Accordingly, one can easily imagine that a slow scan of a fast-moving object would result in a blurred image of the object, resulting in low spatial resolution [128,129]. Biological molecules exhibit translational diffusion, rotational diffusion and conformational changes related to their functions, all of which affect the appearance of observed objects (Fig. 5) and therefore the apparent spatial resolution of the AFM image. Thus, improving the temporal resolution of AFM is key to observing dynamic and mobile biological molecules and clearly resolving their detailed features.

AFM, atomic force microscopy.

This concept is demonstrated by a study that includes HS-AFM imaging of an intrinsically disordered protein. It was demonstrated that the disordered regions are not visible at 1 fps, only slightly visible at 2 fps, visible with their end regions appearing blurry at 5 fps and clearly visible for the entire length at 10 fps [130]. Indeed, using a high temporal resolution to obtain a high apparent spatial resolution, HS-AFM has been used to determine the stoichiometry of protein complexes that have not been determined by other methods [35,131–134]. Thus, improving the temporal resolution of AFM concomitantly improves the apparent spatial resolution of images.

### Further improvements of HS-AFM

**Fig. 6.**
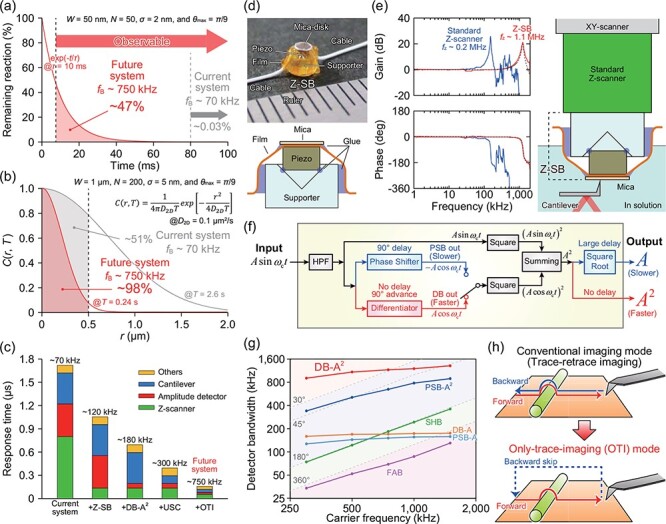
Current efforts to improve the temporal resolution of HS-AFM. (a) Relationship between an enzymatic reaction and the temporal resolution of current and future HS-AFM. The enzymatic reaction was assumed to be a first-order reaction with a time constant of 10 ms. (b) Relationship between the 2D diffusion of a membrane protein on living cells and the temporal resolution of current and future HS-AFM. The probability *C*(*r, T*) represents whether a molecule diffusing on a 2D plane remains in a given observation area of radius *r* after a given time *T*. (c) Summary of response time and feedback bandwidth of various HS-AFM systems. (d) Picture (top) and schematic (bottom) of Z-SB [136]. (e) Left, comparison between the frequency responses of the standard Z-scanner (blue) and Z-SB (red). Right, schematic showing how to install the Z-SB into an HS-AFM system. The Z-SB is attached onto the top of a standard Z-scanner [136]. (f) Circuit diagram of DB and PSB amplitude detector [140]. (g) Detector bandwidth of various amplitude detectors [140]. (h) Comparison between the conventional imaging (trace–retrace imaging) mode and the OTI mode [141].

The power of HS-AFM has been progressively demonstrated by imaging studies on various targets as mentioned earlier. However, there are still a large number of biological processes that cannot be visualized with current HS-AFM systems. This is largely due to the insufficient temporal resolution of HS-AFM. For example, typical enzymatic reactions take place on a time scale shorter than ∼10 ms. However, with a *T*_min_ of ∼80 ms estimated by Eq. (5) under typical imaging conditions (*W* = 50 nm, *N* = 50, *σ* = 2 nm, *f*_B_ = 70 kHz and *θ*_max_ = *π*/9), HS-AFM only captures ∼0.1% of the enzymatic reactions taking place; the remaining ∼99.9% will have completed in the time it takes to acquire a single image (Fig. 6a). In addition, while there are numerous membrane proteins that diffuse on the surface of eukaryotic cells, they have not been observed by AFM at all. This is due to the relationship between the diffusion constant of membrane proteins on the cellular membrane (*D*_2D_ ∼ 0.1 μm^2^/s) [135] and the low temporal resolution *T*_min_ ∼ 2.6 s under typical imaging conditions for living cells (*W* = 1000 nm, *N* = 200, *σ* = 5 nm, *f*_B_ = 70 kHz and *θ*_max_ = *π*/9). About half of the molecules of interest diffuse out of the observation area while taking an image (Fig. 6b), making it impossible to identify and track the same molecule in subsequent images.

2D, two-dimensional; DB, differential-based; USC, ultra-small cantilever; HS-AFM, high-speed atomic force microscopy; OTI, only-trace-imaging; PSB, phase-shift-based; Z-SB, Z-scanner speed-booster; FAB, Fourier analysis–based detector.

Therefore, further improvement of the temporal resolution of HS-AFM is a key stepping stone toward visualizing a wider range of biological phenomena. As mentioned previously, the speed performance of AFM is determined by *f*_B_, which is correlated to the sum of the response times of the devices contained in the feedback loop for maintaining the tip–sample interaction force (Fig. 6c). To substantially increase *f*_B_, all devices need to be improved. Here, we summarize recent efforts to that end.

First, we developed a new Z-scanner named ‘Z-scanner speed-booster’ (Z-SB) with *f*_z_ >1.1 MHz, corresponding to *τ*_z_ of ∼0.14 μs (Fig. 6d and e) [136]. In the mechanical design, a small piezo actuator is supported at its bottom four vertices on a cone-like hollow (Fig. 6d), allowing *f*_z_ to remain as high as that of the piezo actuator in free vibration. As Z-SB is light and compact, it can be integrated to improve the performance of a sample-scanning AFM system without modifying the original Z-scanner (Fig. 6e right). By combining Z-SB with the dual Z-scanner system [137,138], short timescale dynamic molecular events occurring on the surfaces of organelles and cells can be imaged with higher temporal resolution.

Regarding the amplitude detector, the sample-and-hold-based method with Δ*φ* of *π* (i.e. 180°) used to be the fastest type [14]. To reduce Δ*φ*, Miyagi and Scheuring introduced a novel analog amplitude detector based on trigonometric calculation [139], in which a phase shifter was used to obtain a signal with a phase delay of 90° from the input signal: this is known as the phase-shift based (PSB) method. Theoretically, a PSB detector is expected to reach Δ*φ* of 30°. However, due to the circuit latency, the actual Δ*φ* was ∼138° at *f*_c_ of 0.5 MHz which corresponds to *τ*_amp_ of ∼0.77 μs.

To further reduce Δ*φ*, we developed a new amplitude detector using a differential-based (DB) detection algorithm (Fig. 6f) [140], which theoretically has no intrinsic latency, by modifying the PSB algorithm. As this detector generates squared amplitude (*A*^2^), we call this detector DB-A^2^. Although true zero latency performance could not be observed experimentally due to the analog circuit latency, we found that at *f*_c_ < 1 MHz, the detector bandwidth surpassed *f*_c_ (corresponding to the 45° line) and even the theoretical PSB bandwidth of the 30° line (Fig. 6g). At *f*_c_ of 0.5 MHz, Δ*φ* is calculated to be ∼20°, corresponding to *τ*_amp_ of ∼0.11 μs. The square root operation needed in conventional amplitude detectors was determined to be a significant bottleneck (Fig. 6g). In addition to the speed advantage incurred by eliminating the square root operation, the use of the *A*^2^ signal makes the amplitude distance dependence steeper, which contributes to reducing the invasiveness of AFM imaging (see the supplemental material in ref [140]). Finally, by employing faster operational amplifiers, the detector bandwidth could be further improved to at least 2 MHz corresponding to *τ*_amp_ of ∼0.06 μs independent of *f*_c_.

The remaining challenge is to speed up the cantilever. To achieve higher *f*_c_ while keeping *k*_c_ as small as the current model (∼0.2 N/m), further miniaturization of the cantilever is required. Our preliminary experiment suggests that the deflection signal of a cantilever with a length of 2 μm and a width of 0.75 μm can be measured using the current OBD detection system with a slight modification. If the thickness could be reduced to 50 nm, it would be possible to realize an ultra-small cantilever with *f*_c_ of ∼4.5 MHz in liquid and *k*_c_ of ∼0.2 N/m whose *τ*_c_ corresponds to ∼0.11 μs assuming *Q*_c_ of 1.5.

Successfully combining these improvements could yield an HS-AFM system with a 4-fold improved *f*_B_ of ∼300 kHz for low height samples (Fig. 6c), assuming the following parameters: *τ*_c_ of ∼0.11 µs using a cantilever with *f*_c_ ∼ 4.5 MHz and *Q*_c_ ∼ 1.5 in liquid, *τ*_amp_ of ∼0.06 µs using an advanced DB-A^2^, *τ*_z_ of ∼0.14 µs using Z-SB with *f*_z_ ∼ 1.1 MHz and *Q*_z_ ∼ 0.5, and *τ*_d_ of ∼0.1 µs.

Another opportunity for optimization was found in the relative movement of the tip during scanning. Retrace imaging during backward X-scanning as the tip returns to the start of each scan line produces larger feedback error than trace imaging (i.e. forward X-scanning) because of the different torques exerted on the probe tip by the sample. In light of this, the only-trace-imaging (OTI) mode, which eliminates the retrace scan by lifting the tip away from the surface during the retrace, was introduced (Fig. 6h) [141]. Surprisingly, the OTI mode was found to improve *T*_min_ by up to ∼2.5-fold compared to the conventional trace–retrace imaging mode because the elimination of the retrace scan allows the tip to return to the start of the line at higher speeds, while simultaneously reducing the tip–sample contact time and thereby reducing the invasiveness of AFM imaging. The effective feedback bandwidth of an optimized HS-AFM system with a *f*_B_ of ∼300 kHz could therefore be increased as high as ∼750 kHz through the use of OTI mode imaging, an improvement of >10-fold over the current HS-AFM systems (Fig. 6c). This improved temporal resolution of HS-AFM would greatly expand the range of observable biological phenomena (Fig. 6a and b). Implementing the OTI mode requires minor modifications to the software for operating AFM and an extra D/A board that generates a false amplitude signal during the retrace scan [141].

Continuous efforts to improve the spatiotemporal resolution of HS-AFM are necessary. However, the issue of data asynchronicity is inherent in AFM imaging and cannot be eliminated in principle. Other inherent limitations to AFM imaging include the tip convolution effect and the fact that the inside of the sample cannot be observed at all. Remarkably, different groups are beginning to use the methods of computational science and data science to address these issues. By simulating a pseudo AFM image using the atomically resolved structural information of a biological molecule, the correlation between the experimental AFM image and the theoretical model can be analyzed, providing insight into the molecule’s internal structure [16,142–146]. In addition, a sequential Bayesian data assimilation approach was adopted to address the issue of data asynchronicity in AFM [147–150]. This approach reduced image distortion and noise caused by data asynchronicity [147] and demonstrated that the detailed behaviors of biological molecules including their interiors can be analyzed far beyond the limits defined by the spatiotemporal resolution of HS-AFM [148–150]. Moreover, a method based on a modern machine learning approach was recently developed to remove the tip convolution effect as well as the multi-tip effect [151]. These computational methods could play an important role in extracting additional useful information from HS-AFM data.

## Concluding remarks

Recent advances in structural biology have yielded numerous beautiful albeit static structures at atomic resolution [152]. In addition, time-resolved X-ray crystallography and cryo-electron microscopy have been applied to resolve structures over a time course [153–155]. However, the data are not gathered in real time and consist of ensemble averaged structures encompassing many classes of conformations. In contrast, HS-AFM can visualize the structural changes that occur in a single molecule in real time and real space, allowing a deeper and more direct understanding of the functional mechanism of biological molecules. Therefore, HS-AFM continues to play an important and unique role as a technique in structural biology even given the current limits to its spatiotemporal resolution. HS-AFM remains an advantageous technique for studying heterogeneous, highly fluctuating biological samples; moreover, ongoing development of the microscope components and improvements in spatiotemporal resolution promise to expand the range of observable phenomena. Further innovations, including real-time temperature control [156,157], force modulation [158,159], uneven substrates [33,160] and tip-scanning HS-AFM combined with fluorescence microscopy [161,162], promise to open even more frontiers in single-molecule biology.

Here, we describe how the spatiotemporal resolution of HS-AFM is determined. As with any experimental technique, it is important to be familiar with the principle and methods for assessing performance in order to obtain and interpret high-quality data. We hope that this review will serve to further stimulate the study of biological molecules by HS-AFM.
